# Retinoic Acid Activity in Undifferentiated Neural Progenitors Is Sufficient to Fulfill Its Role in Restricting *Fgf8* Expression for Somitogenesis

**DOI:** 10.1371/journal.pone.0137894

**Published:** 2015-09-14

**Authors:** Thomas J. Cunningham, Thomas Brade, Lisa L. Sandell, Mark Lewandoski, Paul A. Trainor, Alexandre Colas, Mark Mercola, Gregg Duester

**Affiliations:** 1 Development, Aging, and Regeneration Program, Sanford Burnham Prebys Medical Discovery Institute, La Jolla, California, United States of America; 2 Stowers Institute for Medical Research, Kansas City, Missouri, United States of America; 3 Department of Anatomy and Cell Biology, University of Kansas Medical Center, Kansas City, Kansas, United States of America; 4 Department of Molecular, Cellular, and Craniofacial Biology, University of Louisville, Louisville, Kentucky, United States of America; 5 Laboratory of Cancer and Developmental Biology, National Cancer Institute, National Institutes of Health, Frederick, Maryland, United States of America; 6 Department of Bioengineering, University of California at San Diego, La Jolla, California, United States of America; Instituto Gulbenkian de Ciência, PORTUGAL

## Abstract

Bipotent axial stem cells residing in the caudal epiblast during late gastrulation generate neuroectodermal and presomitic mesodermal progeny that coordinate somitogenesis with neural tube formation, but the mechanism that controls these two fates is not fully understood. Retinoic acid (RA) restricts the anterior extent of caudal fibroblast growth factor 8 (*Fgf8*) expression in both mesoderm and neural plate to control somitogenesis and neurogenesis, however it remains unclear where RA acts to control the spatial expression of caudal *Fgf8*. Here, we found that mouse *Raldh2*-/- embryos, lacking RA synthesis and displaying a consistent small somite defect, exhibited abnormal expression of key markers of axial stem cell progeny, with decreased Sox2+ and Sox1+ neuroectodermal progeny and increased Tbx6+ presomitic mesodermal progeny. The *Raldh2*-/- small somite defect was rescued by treatment with an FGF receptor antagonist. *Rdh10* mutants, with a less severe RA synthesis defect, were found to exhibit a small somite defect and anterior expansion of caudal *Fgf8* expression only for somites 1–6, with normal somite size and *Fgf8* expression thereafter. *Rdh10* mutants were found to lack RA activity during the early phase when somites are small, but at the 6-somite stage RA activity was detected in neural plate although not in presomitic mesoderm. Expression of a dominant-negative RA receptor in mesoderm eliminated RA activity in presomitic mesoderm but did not affect somitogenesis. Thus, RA activity in the neural plate is sufficient to prevent anterior expansion of caudal *Fgf8* expression associated with a small somite defect. Our studies provide evidence that RA restriction of *Fgf8* expression in undifferentiated neural progenitors stimulates neurogenesis while also restricting the anterior extent of the mesodermal *Fgf8* mRNA gradient that controls somite size, providing new insight into the mechanism that coordinates somitogenesis with neurogenesis.

## Introduction

Knowledge of how stem cells produce differentiated progeny is essential for understanding organogenesis and for realizing the full potential of stem cells as therapeutic agents. In this regard, an understanding of how extrinsic signals, such as retinoic acid (RA) and fibroblast growth factor (FGF), normally regulate stem cell differentiation in vivo is of paramount importance for elucidating effective stem cell treatment regimens that efficiently generate specialized cells. Treatment of stem/progenitor cells in vitro with supraphysiological levels of RA (1–10 micromolar) has for many years been used to induce differentiation in various directions [1,2]. However, little is known about how endogenous RA, normally present at 1–100 nM in various mammalian embryonic or adult tissues [3,4,5,6], controls differentiation of endogenous stem cells in embryos or adults. Thus, knowledge of how endogenous RA controls stem cell populations in vivo is needed to provide guidance on how RA can be used most effectively for therapeutic stem cell treatments.

Recent studies have demonstrated that an endogenous axial (neuromesodermal) stem cell population in vertebrate embryos is an excellent model for investigating signaling mechanisms that normally control stem cell differentiation in vivo [7]. Bipotent axial stem cells expressing *T* (*Brachyury*) and *Sox2* reside in the caudal lateral epiblast lying on each side of the primitive streak [8,9,10]. Axial stem cells differentiate into either neuroectodermal or presomitic mesodermal progeny in a coordinated manner to generate the neural tube and somites that comprise much of the trunk and tail regions [11,12]. Axial stem cells that enter the primitive streak undergo epithelial-to-mesenchymal transition and differentiate into presomitic mesoderm progenitors expressing *Tbx6*, while cells that stay in the anterior region of the caudal epiblast epithelial layer differentiate to neural plate expressing high levels of *Sox2* as the body axis extends. The fate of axial stem cells during differentiation is determined by the decision to express either *Sox2* needed for neural fate or *Tbx6* that helps stimulate presomitic mesodermal fate by repressing *Sox2* [13]. Consistent with this idea, *Tbx6* loss-of-function results in the formation of ectopic neural tubes at the location where somites normally form [14]. Caudal Wnt and FGF signals are required to maintain progenitors (including axial stem cells) that promote body axis extension [8,9,15,16,17,18,19,20]. Wnt and FGF have also been associated with priming of the *Sox2* N1-enhancer to allow moderate expression of *Sox2* in the caudal epiblast (where axial stem cells reside) which is later up-regulated in neural progeny [13]. However, mechanisms that govern this signaling network in order to determine the correct proportion of axial stem cell fates and proper formation of tissues remain unclear.

RA functions as a ligand for widely-expressed nuclear RA receptors (RARa, RARb, RARg) that bind as RAR/RXR heterodimers to RA response elements (RAREs) near target genes [21]. RA is synthesized by mesodermal progeny of the axial stem cell niche through the actions of retinol dehydrogenase 10 (RDH10) that metabolizes retinol (vitamin A) to retinaldehyde followed by retinaldehyde dehydrogenase 2 (RALDH2; ALDH1A2) that metabolizes retinaldehyde to RA which functions as a ligand for RARs [22,23]. Loss of RA synthesis in avian vitamin A deficient embryos and *Raldh2*-/- embryos results in loss of posterior neural differentiation, expansion of presomitic mesoderm along the anteroposterior axis, smaller somite size associated with shortening along the anteroposterior axis of the segmented domain, and premature termination of body axis extension [24,25,26,27]. Excess RA also results in axial truncation, but this is normally prevented by caudal expression of *Cyp26a1* (encoding a RA-degrading enzyme) that is induced by Brachyury (T) under the control of Wnt and FGF signaling [28,29]. As loss of RA results in ectopic anterior expansion of caudal *Fgf8* expression, it has been suggested that RA may control posterior neurogenesis and somitogenesis by antagonizing caudal FGF signaling [24,26,27,30]. Treatment of chick embryos with RA or an RA synthesis inhibitor has been reported to affect not only caudal *Fgf8* expression but also the balance of *Sox2/Tbx6* expression in caudal progenitors at the tailbud stage during termination of body axis extension [9]. Studies on the mechanism of *Fgf8* repression by RA found that during movement of cells from the caudal progenitor zone to the developing trunk, the *Fgf8* chromosomal locus within the nucleus becomes situated more peripherally (a location associated with repression), but this shift to the nuclear periphery was not observed in *Raldh2*-/- embryos [31]. In addition, analysis of transgenes carrying *Fgf8* regulatory elements has shown that a conserved upstream RARE functions to restrict *Fgf8* expression to the caudal progenitor zone of mouse embryos, showing that RA can directly repress *Fgf8* at the transcriptional level [32]. However, it remains unclear in which tissues RA acts to restrict caudal *Fgf8* expression.

Also, in order to understand the mechanism of RA-FGF antagonism in somitogenesis, another important concept to incorporate is the discovery that caudal *Fgf8* transcription occurs in caudal undifferentiated cells but not in more mature progenitors lying anteriorly including the anterior presomitic mesoderm and neural plate [33]; *Fgf8* exon and intron in situ hybridization probes were used to show that anterior presomitic mesoderm displays *Fgf8* mRNA but not *Fgf8* hnRNA primary transcripts, whereas caudal undifferentiated cells exhibited both *Fgf8* mRNA and hnRNA [33]. Although *Fgf8* transcription is turned off in more anterior progenitors, *Fgf8* mRNA persists in these cells to establish a caudal-high to anterior-low gradient of *Fgf8* mRNA and FGF8 protein activity that enacts a gradient of random cell motility and dictates the somite formation wavefront where FGF8 reaches a low anterior signaling threshold [18,19,33,34].

Here, we use RA deficient mouse embryos to show that RA activity in undifferentiated neural progenitors is sufficient to restrict caudal *Fgf8* expression in both neuroectoderm and mesoderm and hence control somite size. Thus, during the process of restricting *Fgf8* expression to control neurogenesis in the axial stem cell niche, RA also restricts the anterior extent of the mesodermal *Fgf8* mRNA gradient to control somite size. Our studies provide new insight into the molecular network that coordinates somitogenesis with neurogenesis.

## Results

### RA deficiency in mouse results in abnormal gene expression in the axial stem cell niche

**Fig 1:**
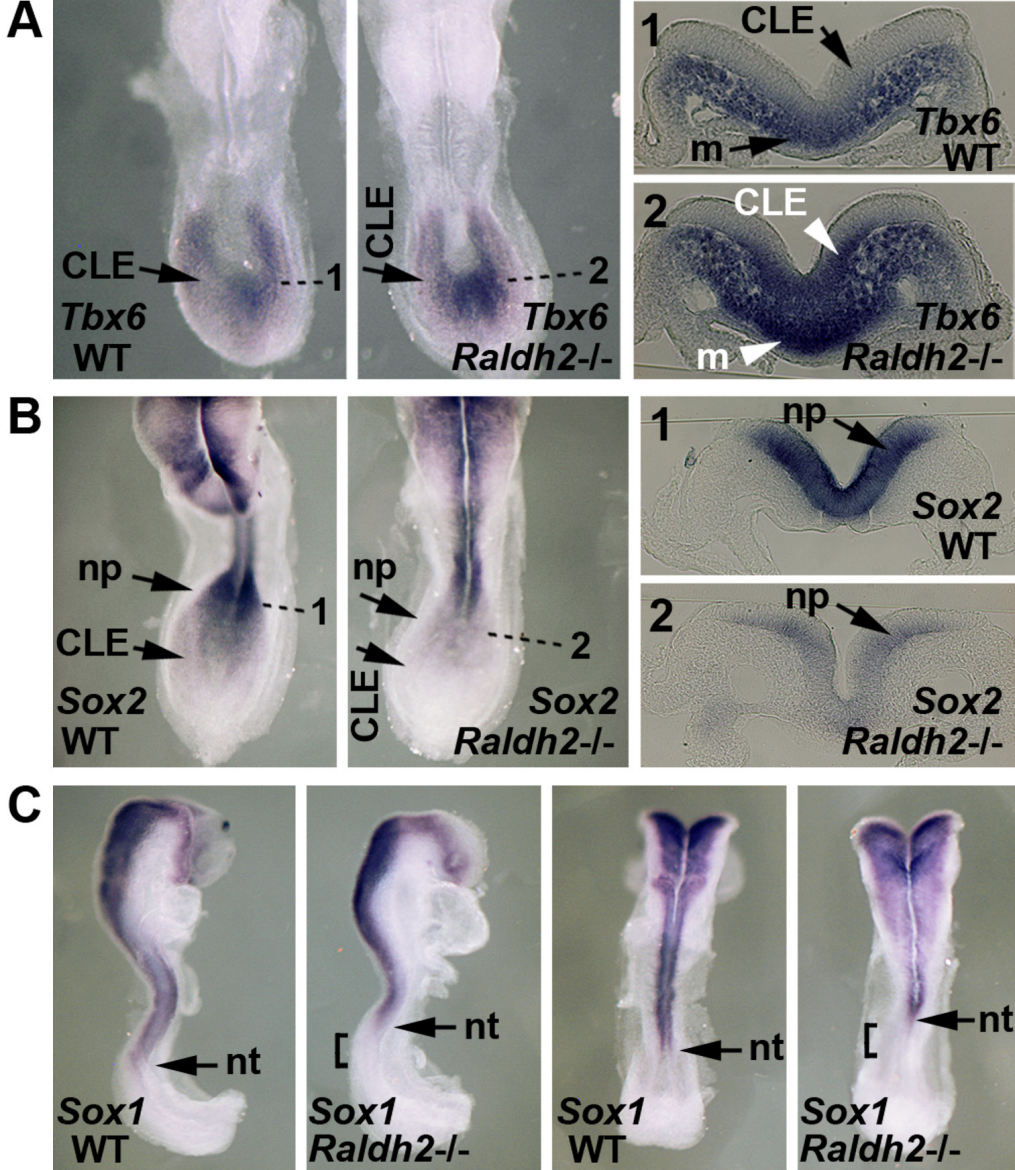
Loss of RA disrupts expression of axial stem cell niche markers. Shown is a comparison of E8.25 (5-somite) wild-type (WT) and *Raldh2*-/- embryos. (A) *Tbx6* mRNA; dotted lines indicate transverse sections through whole-mount stained embryos showing that loss of RA results in the appearance of ectopic *Tbx6* expression in the caudal lateral epiblast (CLE) and expansion of Tbx6+ presomitic mesoderm along the dorsoventral axis. (B) *Sox2* mRNA; dotted lines indicate transverse sections showing that loss of RA down-regulates *Sox2* expression in neural plate (np); also, down-regulation in CLE is observed in whole-mount. (C) *Sox1* mRNA; the bar shows that loss of RA results in loss of *Sox1* expression in posterior neural tube (nt) adjoining the neural plate.

We used genetic loss-of-function studies to explore whether loss of RA synthesis in mouse affects differentiation of axial stem cells needed to generate the body axis. During normal mouse development, *Sox2* (needed for neural fate) is expressed at moderate levels in caudal lateral epiblast (CLE) and at high levels in neural plate progeny located just anterior to the CLE in the same epithelial plane. In contrast, *Tbx6* (that stimulates presomitic mesodermal fate by repressing *Sox2*) is expressed in mesodermal progeny that emerge from the primitive streak near the CLE [13]. We found that E8.25 (5–8 somite) *Raldh2*-/- embryos exhibited ectopic *Tbx6* expression encroaching into the CLE and an expanded domain of Tbx6+ presomitic mesoderm suggesting an increased number of presomitic mesodermal progeny. (Fig 1A; *n* = 3/3). Conversely, *Sox2* expression was decreased in the CLE and neural plate (Fig 1B; *n* = 5/5). This finding is consistent with studies showing that *Tbx6* represses *Sox2* in the axial stem cell niche [13]. In addition, expression of *Sox1* (an early marker of neural progeny) was down-regulated in the posterior-most neural tube but normal more anteriorly, indicating anteriorization of the neural specification boundary, consistent with the lack of *Sox2* upregulation that normally defines the neural plate (Fig 1C; *n* = 3/3). Previous studies reported down-regulation of caudal *Sox2* in RA-deficient embryos, but *Tbx6* and *Sox1* were not examined [35]. Therefore, our additional examination of *Tbx6* and *Sox1* allows insight into axial stem cell regulation. Together, these observations indicate that endogenous RA signaling is required for normal *Sox2*/*Tbx6* expression in the mouse axial stem cell niche, with abnormal *Sox2*/*Tbx6* expression likely contributing to somite axial defects and loss of posterior neurogenesis observed in RA-deficient embryos.

### Defective somitogenesis in RA-deficient embryos is due to ectopic caudal *Fgf8* expression

**Fig 2:**
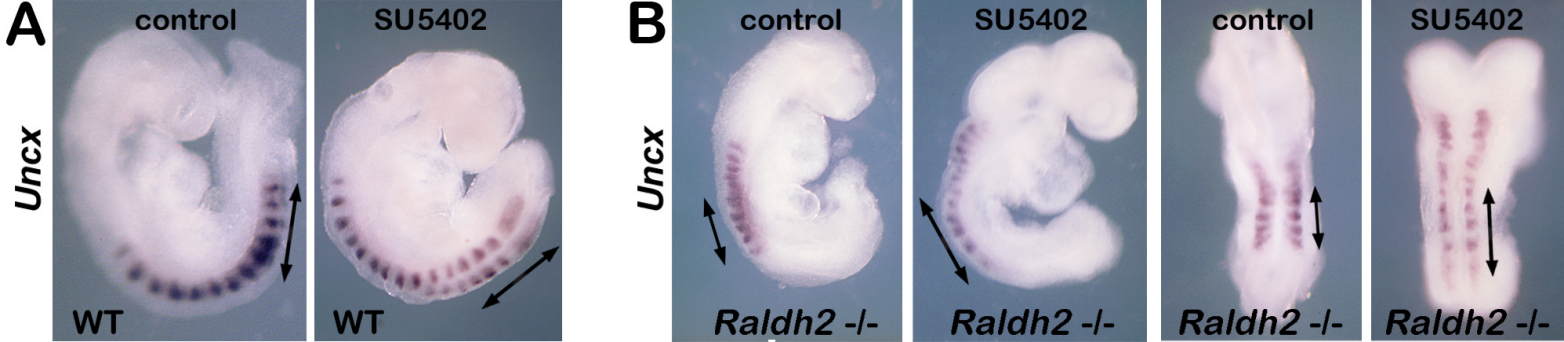
RA antagonism of caudal FGF signaling rescues somitogenesis defect. (A-B) Wild-type (WT) and *Raldh2*-/- embryos at the 6–9 somite stages were cultured for 12 h in the presence of SU5402 (20 micromolar) or DMSO vehicle control, then processed to visualize mRNA for *Uncx* gene expressed in the posterior domain of each somite [26,27] to monitor somite length along the anteroposterior axis. Somite length was compared by measuring the bars bracketing the caudal-most 5 somites generated in culture.

During differentiation of caudal progenitors, FGF signaling becomes gradually down-regulated in both mesodermal and neural progeny due to cessation of *Fgf8* transcription and gradual Fgf8 mRNA decay [33]. Down-regulation of FGF signaling in neural plate cells emerging from the caudal epiblast is required for neurogenesis to proceed normally as the body axis extends [31]. FGF signaling stimulates presomitic mesoderm production through *Tbx6* activation in the primitive streak [15,18], while a posterior (high) to anterior (low) FGF gradient controls a mesodermal random cell motility gradient required for presomitic mesoderm to differentiate into somites during axial elongation [36]. Thus, our observed alterations in *Tbx6/Sox2* expression and somite defects in *Raldh2*-/- embryos may both be explained by expanded caudal *Fgf8* expression that favors Tbx6+ presomitic mesoderm production and maintenance but also disturbs the caudal FGF motility gradient such that the expanded domain of Tbx6+ mesoderm is unable to mature to somite formation due to excess random motility [26,27]. To functionally test the requirement of caudal RA-FGF antagonism for somitogenesis, we cultured wild-type and *Raldh2*-/- embryos in the presence or absence of 20 micromolar SU5402, an inhibitor of FGF receptor activity [37]. We examined embryos for expression of *Uncx* [26,27], a marker for somitic mesodermal progeny of axial stem cells downstream of *Tbx6* expression in presomitic mesoderm. Embryos collected at E8.25 and cultured for 12 hours consistently extended the body axis by five somites (*n* = 8/8 wild-type; *n* = 10/10 *Raldh2*-/-). In control cultures, the newly-generated somites in *Raldh2*-/- embryos were reduced in axial length by 65% versus wild-type (Fig 2A and 2B; *n* = 5/5 for both control and SU5402-treated), recapitulating in vitro the small somite phenotype observed in vivo. SU5402-treated wild-type embryos appeared similar to wild-type controls, indicating that the 20 micromolar concentration used here does not reduce normal FGF signaling enough to affect somitogenesis (Fig 2A; *n* = 4/4 for both control and SU5402-treated). In SU5402-treated *Raldh2*-/- embryos, the newly-generated somites were increased in axial length by 55% versus untreated *Raldh2*-/- controls (Fig 2B; *n* = 5/5 for both control and SU5402-treated). Rescue of the *Raldh2*-/- small somite phenotype following reduction of FGF signaling provides evidence that ectopic caudal FGF signaling observed in *Raldh2*-/- embryos is responsible for the defect.

**Fig 3:**
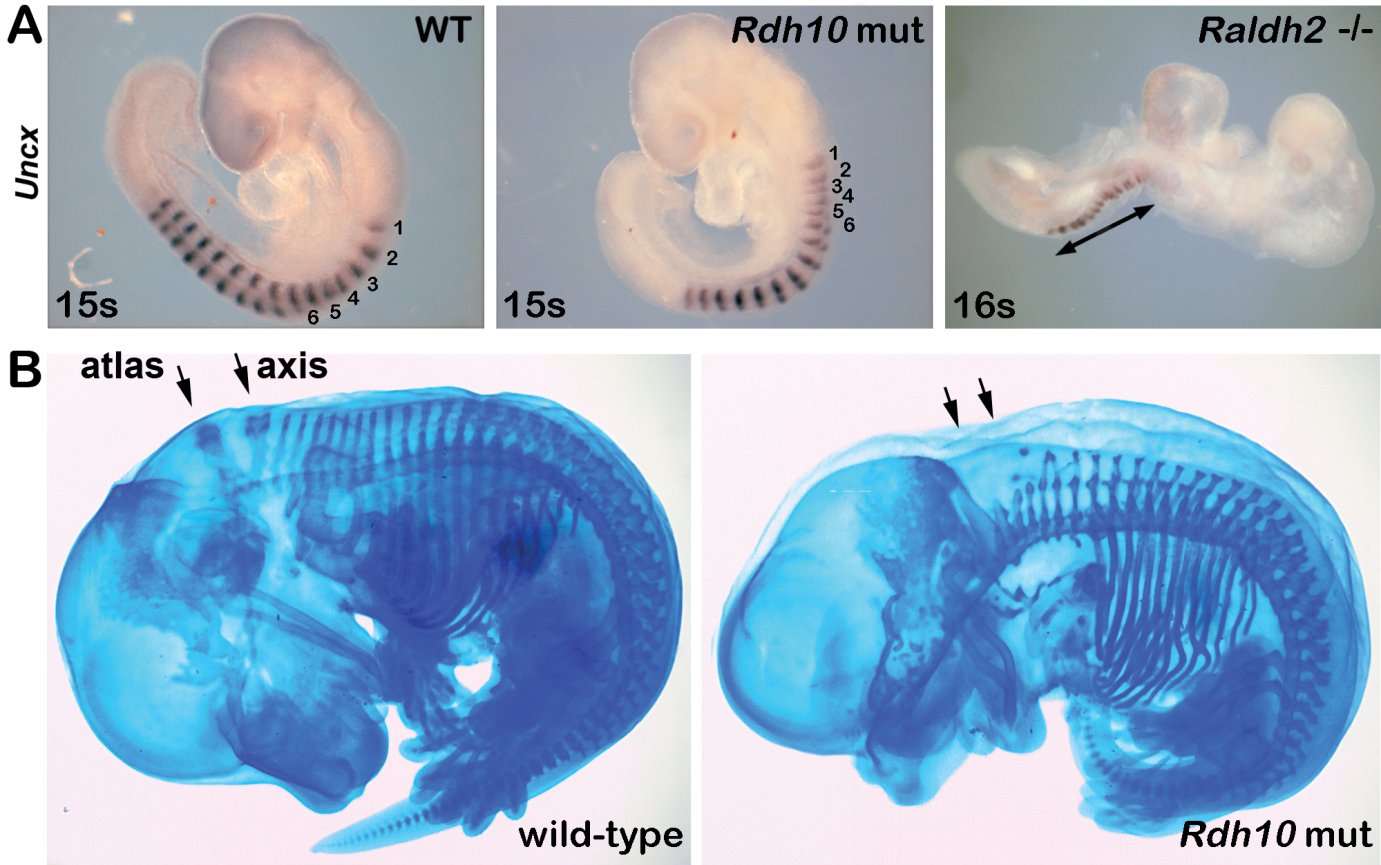
*Rdh10* mutants exhibit a small somite defect during early but not late stages. (A) *Uncx* mRNA in WT, *Rdh10* mutants, and *Raldh2*-/- embryos at the 15–16 somite stage. Numbers marking the first 6 somites of WT and *Rdh10* mutants reveal a temporary shortening of somite size along the anteroposterior axis in the *Rdh10* mutant; arrows mark the region displaying a much larger region of small somites in *Raldh2*-/- embryos. (B) Alcian blue staining of E14.5 wild-type and *Rdh10* mutant embryos was performed; arrows indicate that the mutant lacks the atlas and axis vertebrae derived from somites 5 and 6, respectively.

To gain further insight into caudal RA-FGF antagonism during body axis extension, we examined *Rdh10* ^*trex/trex*^ mice (*Rdh10* mutants) that lack RA activity in somitic mesoderm but retain RA activity in the neural tube and survive longer than *Raldh2*-/- mice [38]. *Rdh10* mutants examined between the 8–15 somite stages exhibited an axial defect in which the first 6 somites were consistently shorter along the anteroposterior axis (axial length between somites 1–6 reduced to 60% of wild-type), whereas somite size returned to normal beginning with somite 7 (Fig 3A; *n* = 8/8). For comparison, analysis of *Raldh2*-/- embryos examined between the 8–16 somite stages demonstrated that all somites were smaller along the anteroposterior axis (Fig 3A; *n* = 11/11). To obtain further insight we examined *Rdh10* mutants at later stages; after E9.5, somites 1–4 normally degenerate and contribute to the occipital bone at the base of the skull, whereas somites 5 and 6 form the atlas and axis vertebrae, respectively [39]. Alcian blue staining of vertebrae at E14.5 revealed that *Rdh10* mutants lack the prominent dorsal extensions normally observed in the atlas and axis vertebra derived from somites 5 and 6, whereas more posterior vertebrae were comparatively normal (Fig 3B; *n* = 2/2). Our observation that the first six somites of *Rdh10* mutants are small but that somite size returns to normal afterwards provides further evidence that anterior trunk mesoderm is developmentally distinct from the posterior trunk, and may require different genetic and signaling networks for its formation as previously described [40,41,42,43]. Together, these observations demonstrate that *Rdh10* mutants undergo an early period where somites appear small, but that somitogenesis appears normalized by the 7-somite stage.

**Fig 4:**
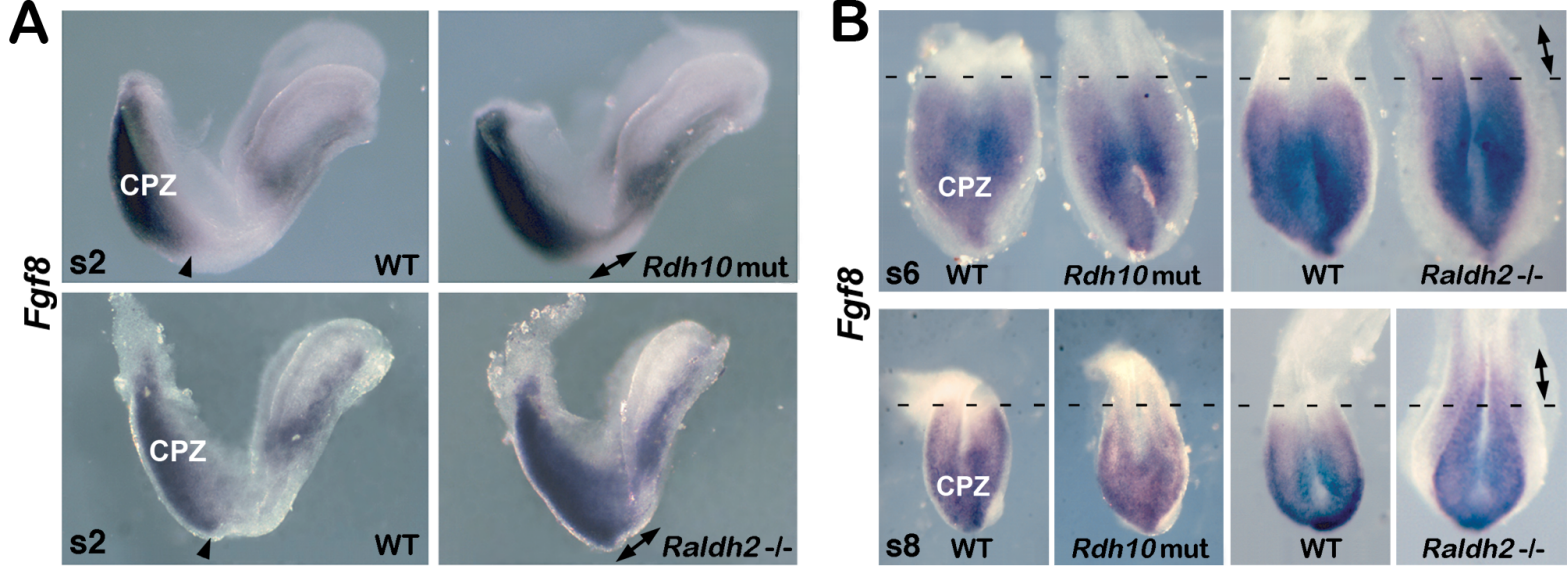
*Rdh10* mutants exhibit ectopic caudal *Fgf8* expression at early but not late stages. (A) *Fgf8* mRNA in 2-somite (2s) embryos. In WT, arrowheads mark the normal anterior border for caudal *Fgf8* expression. In mutants, arrows mark regions of expanded *Fgf8* expression within and anterior to its normal caudal domain. (B) *Fgf8* mRNA in the caudal region (anterior side up) at stages somite-6 (s6) and somite-8 (s8). Dotted lines mark the normal anterior border for caudal *Fgf8* expression in WT. Arrows mark regions of expanded *Fgf8* expression anterior to its normal caudal domain seen for *Raldh2*-/- embryos but not *Rdh10* mutants. CPZ, caudal progenitor zone.

*Rdh10* mutants at the 2–3 somite stages exhibited more intense caudal *Fgf8* expression in the anterior region of its normal caudal epiblast domain as well as some ectopic expression located anterior to its normal domain (*n* = 4/4), similar to *Raldh2*-/- embryos (*n* = 3/3) (Fig 4A). At both the 6-somite and 8-somite stages, the caudal *Fgf8* expression domain in *Rdh10* mutants had returned to normal (*n* = 4/4), whereas *Raldh2*-/- embryos still exhibited ectopic anterior expansion (*n* = 4/4) (Fig 4B). These findings show that during early somite stages when *Rdh10* mutants exhibit a small somite defect, they cannot properly restrict their caudal *Fgf8* expression domain, but caudal *Fgf8* expression is normal by the 6-somite stage at which point somite size returns to normal. Together with our observations above, these findings provide evidence that RA controls both axial stem cell differentiation and somitogenesis by restricting caudal *Fgf8* expression.

### RA signaling in undifferentiated neural progenitors is sufficient to control somitogenesis

**Fig 5:**
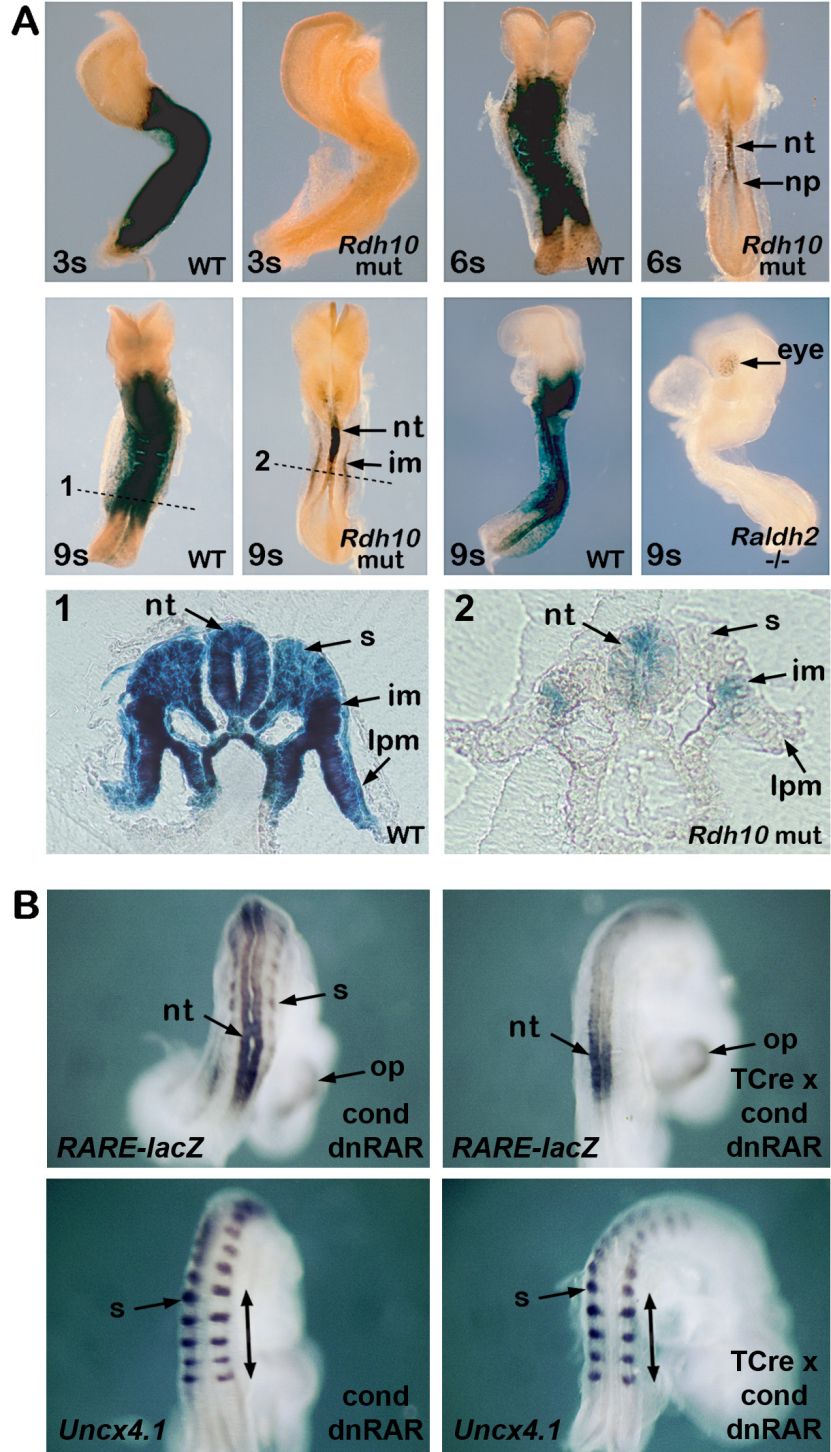
RA signaling in neural plate is sufficient to control somitogenesis. (A) RA activity was visualized in embryos carrying the *RARE-lacZ* RA-reporter transgene using staining for beta-galactosidase activity. At the 3-somite (3s) stage *RARE-lacZ* expression is observed in a wild-type (WT) embryo but not in an *Rdh10* mutant. At the 6s stage *Rdh10* mutants exhibit *RARE-lacZ* expression in posterior neural tube (nt) and neural plate (np). At the 9s stage *Rdh10* mutants exhibit *RARE-lacZ* expression in posterior neuroectoderm and intermediate mesoderm (im); (1–2) transverse sections through the regions marked with dotted lines in 9s embryos; lpm, lateral plate mesoderm; s, somitic mesoderm. At the 9s stage *Raldh2*-/- embryos exhibit no *RARE-lacZ* expression except for weak expression in the eye. (B) Shown are mouse embryos at E8.5 carrying the conditional dominant-negative RAR construct RAR403 (cond dnRAR) or embryos that carry both RAR403 and TCre expressed in mesoderm (TCre x dnRAR). *RARE-lacZ* expression detected by *lacZ* in situ hybridization (to monitor RA activity) shows that TCre-activation of RAR403 prevents RA activity in somitic mesoderm (s) but not neural tube (nt) or optic cup (op). *Uncx* expression shows that loss of mesodermal RA activity does not affect somite size (bars mark last 5 somites generated).

*Fgf8* transcription is detected in caudal undifferentiated cells but not in anterior presomitic mesoderm or neural plate [33]. Our previous studies on *Raldh2*-/- embryos suggested that RA acts in the neural plate and node to control somite bilateral symmetry [27]. However, other studies suggested that RA functions in the presomitic mesoderm to control somitogenesis [44]. To help resolve this controversy, we further examined *Rdh10* mutants which lack mesodermal RA activity but retain neural RA activity at E9.5 [38]. We determined in more detail the onset and location of RA activity in *Rdh10* mutants using *RARE-lacZ* RA-reporter mice [45]. *RARE-lacZ* is a very sensitive RA-reporter as it is induced by 0.25 nM RA, (near the *K*_d_ values reported for RA binding to RARs) as shown previously [46]. We found that *RARE-lacZ* expression initiated late and in a restricted location in *Rdh10* mutants. At the 1–4 somite stages, *RARE-lacZ* expression was not detected in *Rdh10* mutants, showing that *Rdh10* is required to provide retinaldehyde for RA production during these early stages (Fig 5A; *n* = 0/4). At the 6-somite stage, *Rdh10* mutants exhibited *RARE-lacZ* expression in the posterior neuroectoderm extending caudally to the neural plate, but not in other tissues (Fig 5A; *n* = 4/4). *RARE-lacZ* expression in neuroectoderm but not somitic mesoderm was also observed in *Rdh10*-/- null embryos [47], showing that neural RA activity is not due to residual activity of mutant RDH10 protein in *Rdh10* ^*trex/trex*^ embryos but instead is due to another retinol-metabolizing enzyme of unknown identity that contributes to neural RA synthesis beginning at the 6-somite stage (S1 Fig). This unknown activity is not provided by *Cyp1b1*, encoding a P450 enzyme that was previously suggested to contribute to neural RA synthesis [48], as *Rdh10*-/-;*Cyp1b1*-/- double mutants carrying *RARE-lacZ* still exhibited neuroectodermal RA activity and developed similarly to *Rdh10* mutants, plus they exhibited *lacZ* signals in the telencephalon and diencephalon at E10.5 similar to wild-type and *Rdh10* mutants (S1 Fig). At the 9-somite stage, *Rdh10* mutants still exhibited *RARE-lacZ* expression in posterior neuroectoderm but not in presomitic or somitic mesoderm (Fig 5A; *n* = 2/2). For comparison, *Raldh2*-/- embryos at 9–10 somites were devoid of *RARE-lacZ* expression except for weak expression in the eye (Fig 5A; *n* = 3/3). Together, our observations suggest that the transient small somite phenotype observed in *Rdh10* mutants from the 1–6 somite stages is due to a complete lack of early RA synthesis, and that late-occurring RA activity first seen at the 6-somite stage in the neural plate is sufficient to establish normal somitogenesis.

As an independent means of testing whether RA activity in mesoderm is required for normal somitogenesis, we utilized RAR403 mice carrying a dominant-negative RAR (dnRAR) expressed conditionally using Cre/lox technology [49]. When crossed with TCre mice that express Cre only in mesoderm prior to E9.0 [50] and with *RARE-lacZ*, we found that *RARE-lacZ* expression in E8.5 embryos was absent in mesoderm but still present in neuroectoderm (*n* = 3/3) and that somitogenesis remained normal (*n* = 3/3) (Fig 5B). The reciprocal experiment to selectively eliminate RA activity in neural plate but not mesoderm was attempted by crossing RAR403 dnRAR mice with Sox2Cre [51] since *Sox2* is expressed higher in neural plate than caudal epiblast. However, the resultant embryos did not display a clear reduction in neural *RARE-lacZ* (while a reduction was evident in the mesoderm) and somitogenesis as well as overall embryonic development appeared normal (S2 Fig). This experiment would require a different Cre expressed specifically in neural plate, but it is possible that the dnRAR may not fully repress neural RA signaling (by outcompeting endogenous RARs) due to the observation that mouse *RARb* is expressed much higher in the neural tube than the somites [52,53]. However, our observations here with *Rdh10* mutants and dnRAR/TCre embryos combined with the previous observations that *Fgf8* is transcribed in caudal undifferentiated cells but not anterior presomitic mesoderm [33] and that RA can repress caudal *Fgf8* transcription via a RARE [32], suggests that RA transcriptional activity in undifferentiated neural progenitors is sufficient to control the *Fgf8* mRNA gradient for somitogenesis, although we cannot completely rule out a redundant post-transcriptional role for RA in regulating *Fgf8* mRNA in the presomitic mesoderm.

## Discussion

**Fig 6:**
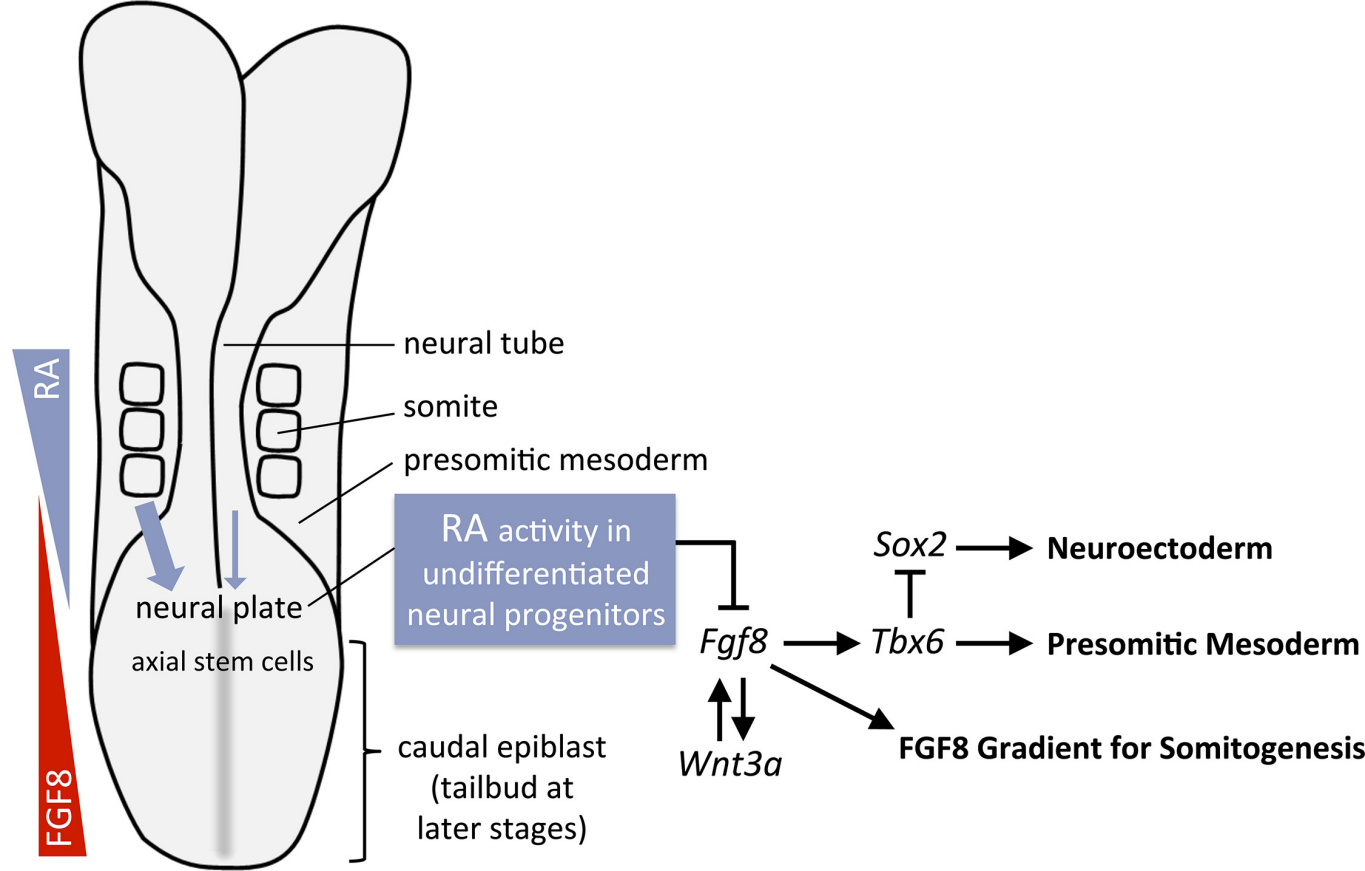
RA control of *Fgf8* coordinates somitogenesis with neurogenesis. Previous studies have shown that RA pushes posterior undifferentiated cells towards differentiation [24,25,26,27]. Our studies support a model in which RA generated by either presomitic mesoderm (high amount—thick blue arrow) or posterior neuroectoderm (low amount—thin blue arrow) functions in undifferentiated neural progenitors to control both neurogenesis and somitogenesis by restricting *Fgf8* expression. RA participates in a gene regulatory network in which activation of *Tbx6* in the axial stem cell niche is dependent on signaling controlled by *Fgf8* and *Wnt3a*, with *Wnt3a* and *Fgf8* participating in an autoregulatory loop. RA restricts *Fgf8* expression to provide the correct amount of both *Tbx6* and *Sox2* expression, with *Sox2* being repressed by *Tbx6* during generation of mesodermal progeny. By limiting *Fgf8* expression in undifferentiated neural progenitors, RA also establishes the anterior boundary of the *Fgf8* mRNA gradient in presomitic mesoderm that controls somitogenesis.

The studies reported here provide important new insight into how extrinsic signals coordinate somitogenesis with neurogenesis. Previous studies demonstrated that axial stem cell differentiation normally results in a balance between progeny that express *Sox2* (required for neuroectodermal fate) and progeny that express *Tbx6* (required for mesodermal fate), and that the mechanism requires indirect repression of *Sox2* by *Tbx6* [13]. Also, expression of *Sox2* or *Sox3* in the caudal lateral epiblast is required to prevent excessive production of mesodermal progeny from axial stem cells [54]. Activation of *Tbx6* in the axial stem cell niche is controlled by caudal FGF and Wnt signaling through a signaling loop in which FGF and Wnt positively activate each other [8,9,15,16,17,18,19]. In mouse, those studies revealed that FGF receptor 1 and caudal *Fgf8* expression are required to maintain caudal expression of *Wnt3a*, which in turn is required to maintain caudal *Fgf8* expression, with this feedback loop being required for *Tbx6* expression during mesoderm induction. Our studies here support and further extend previous studies showing that RA is required to restrict the anterior extent of caudal *Fgf8* expression in chick and mouse [24,26,27,30] and another study in chick embryos showing that RA effects caudal *Sox2/Tbx6* expression at the tailbud stage when body axis extension is terminated [9]. Our *Raldh2*, *Rdh10*, and dnRAR/TCre genetic studies in mouse support a model in which progeny of the axial stem cell population produce a diffusible RA signal that acts in undifferentiated neural progenitors to restrict the anterior extent of caudal *Fgf8* expression in order to control a gene regulatory network underlying both neurogenesis and somitogenesis (Fig 6). As a result of this ability to restrict *Fgf8* expression, RA signaling functions as a rheostat to dial in the correct amount of caudal FGF signaling needed to coordinate somitogenesis with neurogenesis. This mechanism ensures that generation of presomitic mesodermal progeny is restricted and that mesodermal motility is appropriately graded to allow controlled differentiation into somites, while normal differentiation of neural progeny (i.e. *Sox1* activation) is also permitted. Our studies thus reveal that the function of RA during body axis extension is that of a progeny-generated signal regulating differentiation of its parent stem cell population.

Our findings suggest a mechanism for how loss of RA in *Raldh2*-/- embryos results in smaller somites, an expanded region of presomitic mesoderm, and ultimately premature cessation of body axis elongation [25,26,27]. We propose that ectopic *Fgf8* expression observed in *Raldh2*-/- embryos not only disturbs the caudal-high motility gradient needed for normal somitogenesis and body axis extension [36], but also expands *Tbx6* expression in the axial stem cell niche that may inhibit somite formation as this promotes a migratory presomitic mesodermal fate that must be efficiently down-regulated to form somites [55]; thus fewer cells contribute to somites resulting in smaller somites accompanied by expansion of unsegmented presomitic mesoderm caudally. In addition, we propose that lower *Sox2* expression in the caudal epiblast and neural plate of *Raldh2*-/- embryos may be due to ectopic expression of *Tbx6* in the caudal epiblast that may repress *Sox2*, leading to loss of axial stem cell maintenance. This general failure to coordinately form mature body axis tissues, born out of an imbalance in the axial stem cell niche due to excessive FGF signaling, may explain premature cessation of body axis elongation in RA-deficient embryos.

Previous studies have shown that *Fgf8* transcription is detected caudally in undifferentiated cells but not in anterior presomitic mesoderm or neural plate; after presomitic mesoderm cells exit the caudal undifferentiated cell population and turn off *Fgf8* transcription, they retain *Fgf8* mRNA which persists for some time and forms a caudal-high gradient of *Fgf8* mRNA that in presomitic mesoderm terminates anteriorly near the somite formation boundary [33]. Additionally, our previous studies have demonstrated that RA can repress caudal *Fgf8* expression directly at the transcriptional level [7]. As our studies here show that RA activity in undifferentiated neural progenitors is sufficient to properly regulate caudal *Fgf8* expression and somite size, RA is not required to act in presomitic mesoderm to control the anteroposterior positioning of the mesodermal *Fgf8* mRNA gradient. Our conclusions are thus in contrast to previous studies in *Xenopus* suggesting that RA functions in presomitic mesoderm to control somitogenesis [44]; those studies showed that RA treatment can induce anterior presomitic mesodermal expression of *Xenopus Mesp2*, a gene required to generate the somite formation boundary, and it was reported that the *Xenopus Mesp2* gene has a RARE. However, later studies in mouse *Raldh2*-/- embryos showed that endogenous RA is not required for expression of *Mesp2* [27], demonstrating that results of RA treatment do not always correlate with endogenous RA function. Also, as vertebrate genomes contain more than 10,000 RAREs, many of which may be non-functional [21], it is possible that the genomic RARE located near *Xenopus Mesp2* may not be required in vivo although it can function in a transgenic assay. Likewise, although studies on the *Fgf8* RARE in transgenic mice suggest that RA can directly repress caudal *Fgf8* [32], further studies will be needed to determine whether RA directly represses transcription of the genomic *Fgf8* locus solely through this RARE or whether another molecular mechanism also exists to restrict caudal *Fgf8* expression.

The knowledge gained by our studies provides new mechanistic insight into the molecular networks controlling robust differentiation of stem/progenitor cells into specialized cells. Awareness of how diffusible RA and FGF signals normally interact in vivo to control stem/progenitor cell differentiation will aid efforts to generate differentiated cells useful in therapeutic applications.

## Materials and Methods

### Mouse mutants and transgenes

*Rdh10* ^*trex/trex*^ mice (*Rdh10* mutants) were generated via ethylnitrosourea mutagenesis as previously described [38]. *Rdh10* mutant embryos were identified by DNA sequencing of a PCR product overlapping the mutation from analysis of yolk sac DNA [38]. *Rdh10* mutants were mated with *RARE-lacZ* RA-reporter transgenic mice [45], and the offspring were employed throughout the study; *Rdh10*-/- knockout embryos were described previously [47]. *Raldh2*-/- mice carrying *RARE-lacZ* have been previously described [27]. TCre mice were described previously [50]. RAR403 mice carrying a dominant-negative RAR (dnRAR) [49] were obtained from Dr. Shanthini Sockanathan (Johns Hopkins University, Baltimore, MD, USA). *Cyp1b1*-/- mice [56] were provided by Dr. Simon John (Jackson Laboratory, Bar Harbor, ME, USA). *Sox2Cre* mice [51] were purchased from The Jackson Laboratory [Tg(Sox2-cre)1Amc/J, stock # 004783]. Genotyping of each strain was performed by PCR analysis of yolk sac DNA. All mouse studies conformed to the regulatory standards adopted by the Institutional Animal Care and Use Committee at the Sanford Burnham Prebys Medical Discovery Institute which approved this study under Animal Welfare Assurance Number A3053-01 (permit #13–003).

### In vitro culture of mouse embryos

Wild-type, *Rdh10* mutant, and *Raldh2*-/- embryos at the 6–9 somite stages were cultured for 12 hr in serum-free (retinoid-free) DMEM/F-12 culture media (Gibco-Life Technologies) in Millicell culture plate inserts (Millipore) at 37°C in 5% CO_2_; treatments included 20 micromolar SU 5402 (Tocris Bioscience) or an equal amount of dimethylsulfoxide vehicle (control). After culture, embryos were stained with X-Gal for 18 hr or processed for in situ hybridization.

### Detection of mRNA, retinoic acid, and cartilage

Detection of mRNA and tissue sectioning was performed by whole mount in situ hybridization as previously described [27]. RA activity was detected in mouse embryos carrying the *RARE-lacZ* transgene by staining 18 hr for beta-galactosidase activity [45]. Alcian blue staining of cartilage was performed as previously described [38].

## Supporting Information

S1 Fig *Rdh10-/-;Cyp1b1-/-* double mutants carrying *RARE-lacZ* still exhibit neuroectodermal RA activity. Wild type, *Cyp1b1*-/-, *Rdh10*-/-, and *Rdh10*-/-;*Cyp1b1*-/- double mutants carrying the *RARE-lacZ* RA-reporter transgene and stained for beta-galactosidase activity at E10.5.
(TIF)

S2 Fig Crossing RAR403 dnRAR mice with Sox2Cre is insufficient to significantly reduce neural RA signaling. Lateral views (upper panels) and dorsal views (lower panels) of E8.5 embryos carrying the *RARE-lacZ* RA-reporter transgene and the dnRAR transgene, or additionally carrying Sox2Cre. Embryos were stained for beta-galactosidase activity; np, neural plate.
(TIF)
