# Supplementary figures and images for: Retinoic Acid Activity in Undifferentiated Neural Progenitors Is Sufficient to Fulfill Its Role in Restricting Fgf8 Expression for Somitogenesis
Source: PLoS One. 2015 Sep 14;10(9):e0137894. doi: 10.1371/journal.pone.0137894 (PMC4569375; doi:10.1371/journal.pone.0137894)

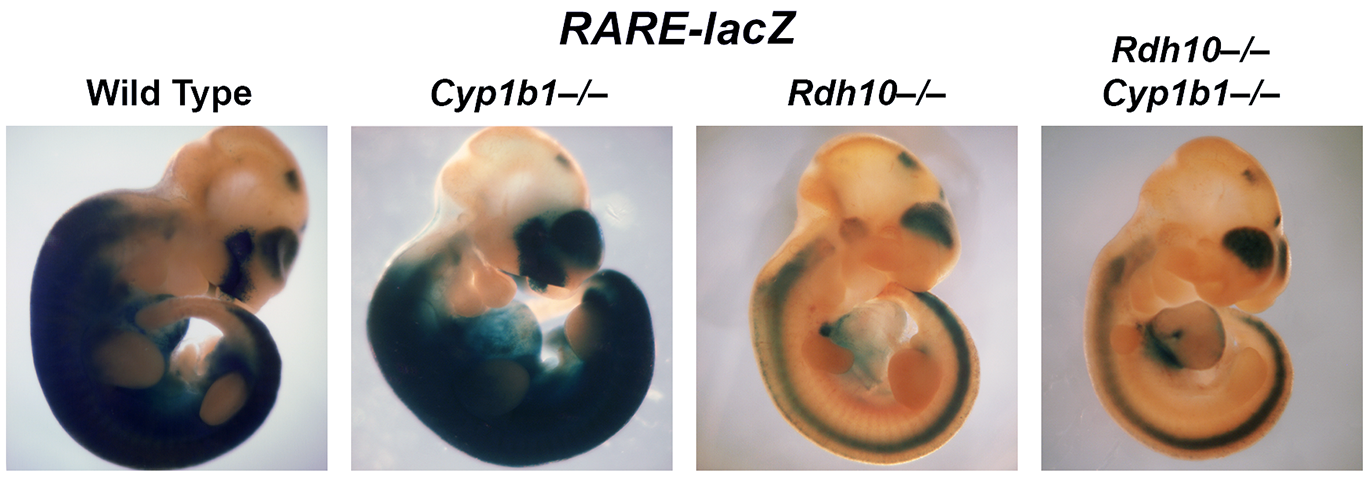

Supplement: S1 Fig — Wild type, Cyp1b1-/-, Rdh10-/-, and Rdh10-/-;Cyp1b1-/- double mutants carrying the RARE-lacZ RA-reporter transgene and stained for beta-galactosidase activity at E10.5. (TIF) [file pone.0137894.s001.tif]

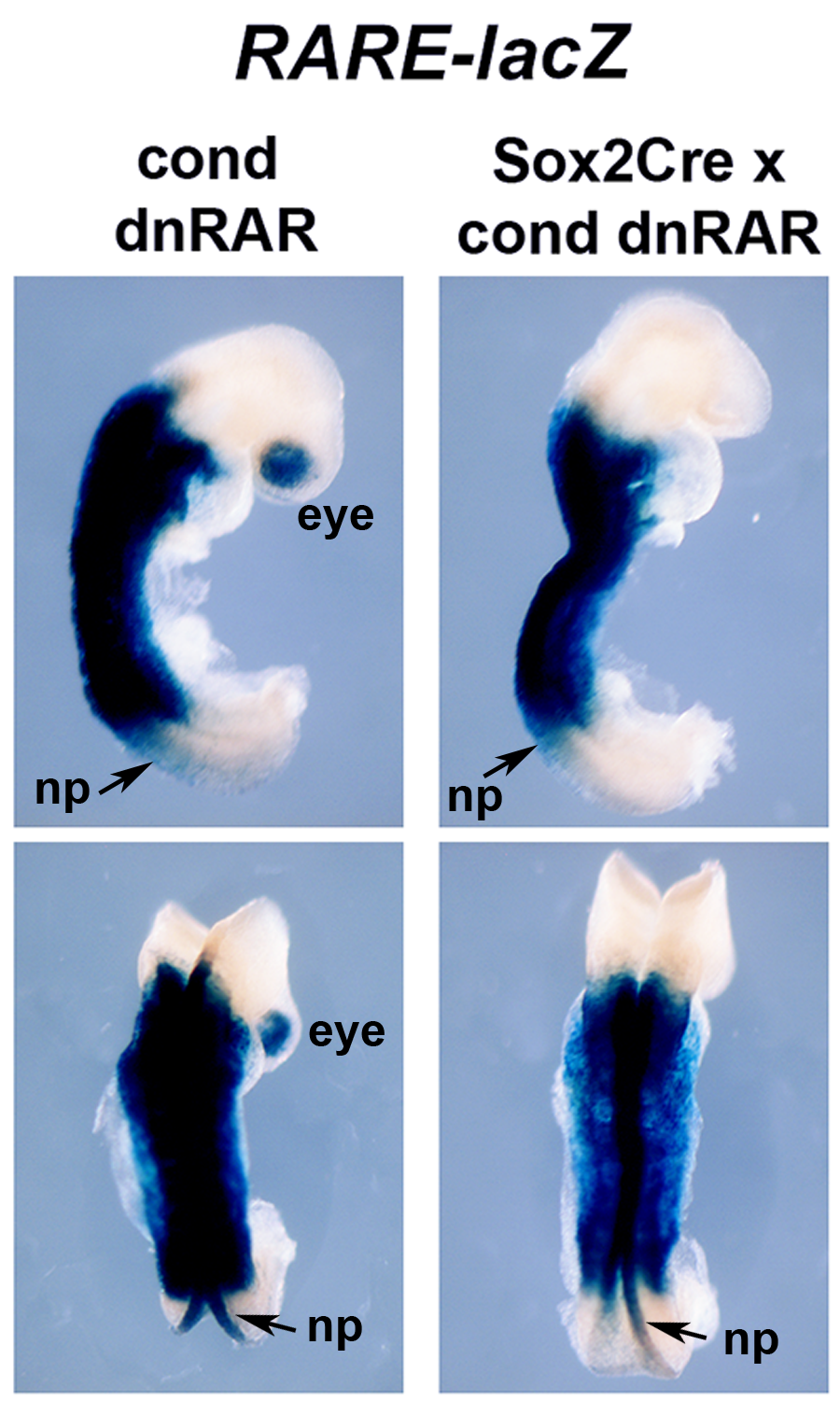

Supplement: S2 Fig — Lateral views (upper panels) and dorsal views (lower panels) of E8.5 embryos carrying the RARE-lacZ RA-reporter transgene and the dnRAR transgene, or additionally carrying Sox2Cre. Embryos were stained for beta-galactosidase activity; np, neural plate. (TIF) [file pone.0137894.s002.tif]
